# Environmental and Health Risk Assessment of Fugitive Dust from Magnesium Slag Yards

**DOI:** 10.3390/toxics13040307

**Published:** 2025-04-15

**Authors:** Jing Hua, Yuanchao Zhao, Yuanzheng Zhang, Yining Zhu, Chang Liu, Fenghe Wang, Xiaowei Xu, Qi Yu

**Affiliations:** 1 Nanjing Institute of Environmental Science, Ministry of Ecology and Environment of China, Nanjing 210042, China; huajing@nies.org (J.H.); zhaoyuanchao@nies.org (Y.Z.); zhangyuanzheng@nies.org (Y.Z.); liuchang@nies.org (C.L.); 2 School of Chemistry and Chemical Engineering, Nanjing University of Science and Technology, Nanjing 210094, China; 222502023@njnu.edu.cn (Y.Z.); wangfenghe@njust.edu.cn (F.W.); 3 Department of Environmental Science, School of Environmental Science and Engineering, Suzhou University of Science and Technology, Suzhou 215009, China; yuqi@usts.edu.cn

**Keywords:** magnesium slag, dust pollution, heavy metals, environmental risk, resource utilization

## Abstract

During the natural cooling process of magnesium slag stockpiles in the open air, the phase transformation of gamma-dicalcium silicate (γ-C_2_S) induces a powdering phenomenon, resulting in the generation of a large amount of PM10 and PM2.5 dust. Based on the dust emission model of stockpiles and the Gaussian dispersion model, combined with the Monte Carlo simulation method, this study conducted a quantitative assessment of the environmental risk of heavy metals (Pb, Cd, Hg, As, Cr(VI)) in dust to the surrounding residential areas. The results show that the enrichment degree of heavy metals in PM2.5 is significantly higher than that in PM10. At a downwind distance of 1000 m, the exceedance multiples of Cr(VI), As, and Cd reach 131.5, 23.6, and 51.8 times, respectively. The total carcinogenic risk (9.2 × 10^−7^) and total non-carcinogenic hazard quotient (0.15) in the residential area are below the limits, but the contribution rates of As and Cd are relatively high. Sensitivity analysis further reveals that the moisture content of the stockpile, dust removal rate, and distance are the key control parameters affecting the environmental risk. Based on the research findings, it is recommended to increase sprinkling frequency, install windbreak nets, and promote magnesium slag utilization to effectively control dust risks.

## 1. Introduction

Magnesium, as an important light metal, is widely used in various fields such as aerospace, automotive manufacturing, electronics, chemicals, and building materials due to its light weight, high strength, and good vibration damping properties [1]. In recent years, with the increasing application of magnesium and its alloys in modern industry, the magnesium production capacity in China has grown rapidly. Currently, the annual output of magnesium resources in China is close to 1 million tons, accounting for about 90% of the global magnesium demand [2]. However, the magnesium smelting process generates a large amount of magnesium slag, and the current recovery rate of magnesium slag is less than 30% [3]. As a result, a large amount of magnesium slag can only be stored in open-air stockpiles. This open-air storage method not only wastes a large amount of land resources [4], but also poses potential environmental pollution and safety hazards, such as landslides and slope failures. Moreover, during the natural cooling process of magnesium slag storage in the open air, the main component γ-C_2_S (dicalcium silicate) undergoes a phase transformation to β-C_2_S, causing volume expansion and resulting in the pulverization of magnesium slag [2]. The pulverized magnesium slag particles are fine and can easily be blown up by the wind [5], forming dust, which, in turn, affects the surrounding ecological and residential environments of the magnesium slag stockpile.

Despite the increasing prominence of environmental issues related to magnesium slag stockpiles, current research and attention have mainly focused on the impact of stockpiles on surrounding soil and groundwater, while the hazards of magnesium slag dust from pulverization have not yet received sufficient attention. Dust not only affects air quality but can also carry heavy metals and other harmful substances, posing a potential threat to the health of nearby residents [6]. Wang et al. [7] found that the mercury content in dust from mining area schools was significantly higher than the local soil background value, with the total mercury content in dust being 2.29 times the background value. Younes Shekarian et al. [8] demonstrated that fine particulate matter with a diameter of less than 10 μm can penetrate deep into the lungs, causing chronic inflammation and even entering the bloodstream to damage the cardiovascular and nervous systems. Moreover, PM2.5 particles, due to their small size and large specific surface area, are more likely to adsorb heavy metals and migrate over long distances, resulting in significantly higher environmental risks than PM10 [9]. However, existing research on the migration patterns of heavy metals in magnesium slag dust and health risk assessment is still insufficient, especially regarding the enrichment effects and long-term exposure risks of PM2.5 particles.

This study focuses on the PM10 and PM2.5 particles generated from the natural pulverization of magnesium slag, combining dust emission models from stockpiles and dust migration and dispersion models. It also employs the Monte Carlo simulation method to conduct an in-depth analysis of the environmental risks and evolution of dust pollution from stockpiles to surrounding residential areas. By quantifying the exceedance multiples of heavy metals, compliance distances, and health risks, this study aims to provide scientific evidence for the environmental management and pollution control of magnesium slag stockpiles and to offer new directions for future research.

## 2. Materials and Methods

### 2.1. Sample Preparation and Analysis

The northwest region of China is the largest magnesium metal production base globally, where the traditional silicothermic method has long been used for magnesium smelting [10]. In this process, approximately 5 to 7 tons of magnesium slag are generated for each ton of magnesium produced [3]. Over decades, a significant number of open-air magnesium slag stockpiles have accumulated in this area. This study collected 80 magnesium slag samples from these stockpiles for analysis. After natural pulverization, the samples were sieved to select particles with diameters of 0~2.5 µm and 2.5~10 µm. These samples were analyzed for a comprehensive list of pollutants including copper, zinc, cadmium, lead, hexavalent chromium, mercury, beryllium, barium, nickel, arsenic, selenium, VOCs, and SVOCs, following the pretreatment and analytical procedures outlined in the standard “Identification Standards for Hazardous Wastes—Identification for Toxic Substance Content” (GB 5085.6-2007) [11].

### 2.2. Characterization of Dust Emission Risk from Stockpiles

#### 2.2.1. Emission Characterization of Pollutants

The primary sources of dust emissions from stockpiles include particulate matter released during operations such as loading, unloading, transportation, and storage, as well as wind erosion. The emission characterization of dust from stockpiles is given by Equation (1) [12,13,14]:

(1) $$ER=V\times k\times 0.0016\times \frac{{\left(\frac{U}{2.2}\right)}^{1.3}}{{\left(\frac{M}{2}\right)}^{1.4}}\times (1-\eta )$$

where ER represents the dust emission concentration in mg/s; k is the particle size multiplier, which is dimensionless; U is the average wind speed at ground level in m/s; M is the moisture content of the material, which is dimensionless; η is the dust removal rate of the stockpile, which is dimensionless; and V is the disturbance rate of the stockpile in m^3^/s.

#### 2.2.2. Migration and Dispersion Characterization of Pollutants

Among the common atmospheric pollutant migration and dispersion models, such as the Gaussian dispersion model, AERMOD model, and ADMS model, the Gaussian dispersion model is widely used due to its simplicity and validation by extensive experimental data. This model assumes that atmospheric pollutants are normally distributed around the centerline of the plume from the pollution source and that the source strength is uniform and continuous. The calculation method is expressed by Equation (2) [15]:

(2) $${PM}_{i}=\frac{ER}{2\pi U\sigma_{y}\sigma_{z}}\;\times \;\exp(-\frac{y^{2}}{2\sigma_{y}^{2}})\;\times \;\{\exp[-\frac{({Z-h)}^{2}}{2\sigma_{z}^{2}}]+\exp[-\frac{({Z+h)}^{2}}{2\sigma_{z}^{2}}]\}\;\times \;\exp(-\frac{gx(\rho -\rho_{a})d^{2}}{18\mu U})$$

where ${PM}_{i}$ represents the concentration of particulate matter at a certain distance from the stockpile in mg/m^3^; $\sigma_{y}$ and $\sigma_{z}$ are the lateral and vertical dispersion coefficients in m; H is the height of the stockpile in m; x, y, and z are the downwind distance, horizontal distance, and vertical distance in m, respectively; ρ is the density of the particulate matter in kg/m^3^; $\rho_{a}$ is the air density in kg/m^3^; d is the particle diameter in m; and μ is the air dynamic viscosity in Pa·s.

#### 2.2.3. Risk Characterization

According to the “Technical guidelines for risk assessment of soil contamination of land for construction” (HJ 25.3-2019) [16], the exposure amount of inhaled particulate matter is calculated using Equation (3), while the carcinogenic risk and non-carcinogenic hazard quotient are calculated using Equations (4) and (5) [16]:

(3) $$Q=\frac{{PM}_{i}\times DAIR\times ED\times PIAF\times EF}{BW\times AT}\times 10^{-6}$$

(4) CR=Q×C×SF

(5) $$HQ=\frac{Q\times C}{RfD\times SAF}$$

where Q represents the intake of inhaled particulate matter in mg/kg-d; DAIR is the daily inhalation rate of an adult in m^3^/d; ED is the exposure duration in years (a); EF is the exposure frequency in days per year (d/a); PIAF is the proportion of inhaled particulate matter retained in the body, which is dimensionless; BW is the body weight of an adult in kg; AT is the average time for (non)carcinogenic effects in days; C is the heavy metal content in particulate matter in mg/kg; SF is the carcinogenic slope factor in (mg/kg-d)^−1^; RFD is the reference dose for inhalation in mg/kg-d; and SAF is the reference dose allocation coefficient for exposure to particulate matter, which is dimensionless.

### 2.3. Uncertainty and Sensitivity Characterization

The Monte Carlo method [17] was used to characterize the impact of parameter uncertainty on the results. The annual average wind speed at ground level was based on the “China Statistical Yearbook 2022”, with an average of 2 m/s and possible variations from 1 to 6 m/s. Stockpile height was measured, with an average of 15 m, ranging from 5.2 to 19 m. Moisture content was measured, with an average of 30%, varying between 21% and 44%. The dust removal rate was set at a default value of 50%, with a range from 0% to 100%. The disturbance rate of the stockpile was set at a default value of 500 m^3^/h, ranging from 250 to 2500 m^3^/h. Particle density was measured, with an average of 1.21 kg/m^3^, ranging from 1.03 to 1.36 kg/m^3^. $\sigma_{y}$ and $\sigma_{z}$ are based on GB/T 3840-1991 [18]. Pollutant contents were determined based on actual measurements. The exposure duration, exposure frequency, body weight, carcinogenic slope factor, reference dose for inhalation, reference dose allocation coefficient for exposure to particulate matter, and proportion of inhaled particulate matter retained in the body, which are related to risk characterization, were adopted from the HJ 25.3-2019.

## 3. Results and Discussion

### 3.1. Characteristics of Dust Pollution from Stockpiled Magnesium Slag

The analysis of pollutant content revealed that organic pollutants were not detected in magnesium slag, and the detection rates of nickel and beryllium were both below 10%, with their concentrations at relatively low levels. Notably, heavy metals such as Pb, Cd, Hg, As, and Cr(VI) were stably detected in all samples, with concentrations significantly higher than those of other heavy metals. Based on these findings, this study identified the five heavy metals mentioned above as the key targets for subsequent environmental risk assessment.

**Figure 1:**
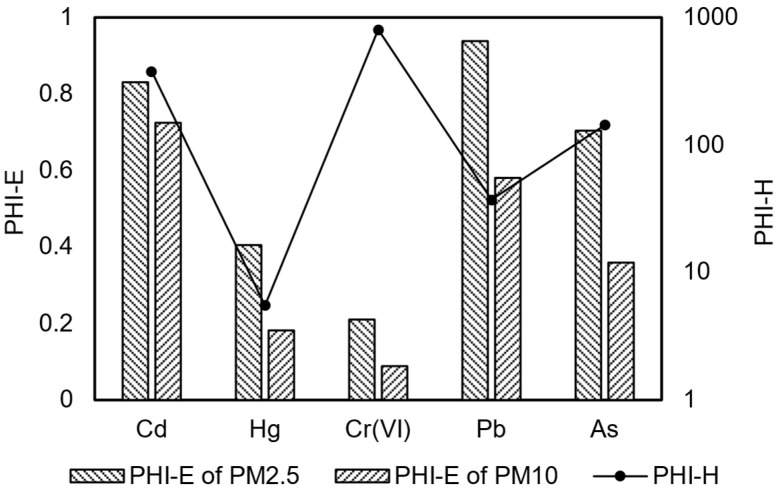
Potential hazard index of heavy metals in dust particles.

To quantify the health risk characteristics of dust pollutants, this study developed a dual-index system of Potential Environmental Hazard Index (PHI-E) and Potential Health Hazard Index (PHI-H). The PHI-E was calculated based on the ratio of heavy metal content in dust particles to the soil environmental quality Class II screening values, while the PHI-H was determined based on the ratio of heavy metal content in dust to air quality standards when the total suspended particulate concentration in the environment is within the standard limit (Figure 1). The results showed that the PHI-E values for PM2.5 and PM10 ranged from 0.21 to 0.94 and from 0.08 to 0.72, respectively, both below the risk threshold of 1. However, the heavy metal content in PM2.5 was significantly higher than that in PM10 for the same elements, consistent with the findings of Wang Jiuzhu et al. [19]. They pointed out that fine particulate matter, due to its larger specific surface area and stronger pollutant adsorption capacity, tends to have higher heavy metal content. Specifically, the PHI-E value for Pb in PM2.5 was the highest, with an average of 650 mg/kg, while the PHI-E value for Cd in PM10 was the largest, with an average of 33 mg/kg. This result is similar to the findings of Chen Qiyue et al. [20], who reported that pollution from Cd and Pb in certain mining area soils posed significant risks to crop growth and human health.

When the concentration of particulate matter in the ambient air reaches the standard limit, the PHI-H analysis showed that the indices for cadmium, mercury, hexavalent chromium, lead, and arsenic reached 376, 5.5, 800, 37, and 143, respectively, all significantly exceeding the limit values. Notably, the PHI-H value for hexavalent chromium was as high as 800, more than 4000 times the PHI-E value. This indicates that although the environmental risk of dust heavy metals to soil and groundwater is relatively low, their health risks to nearby residents through inhalation exposure need to be taken seriously.

### 3.2. Environmental Risk and Evolution of Dust from Stockpiled Magnesium Slag

**Figure 2:**
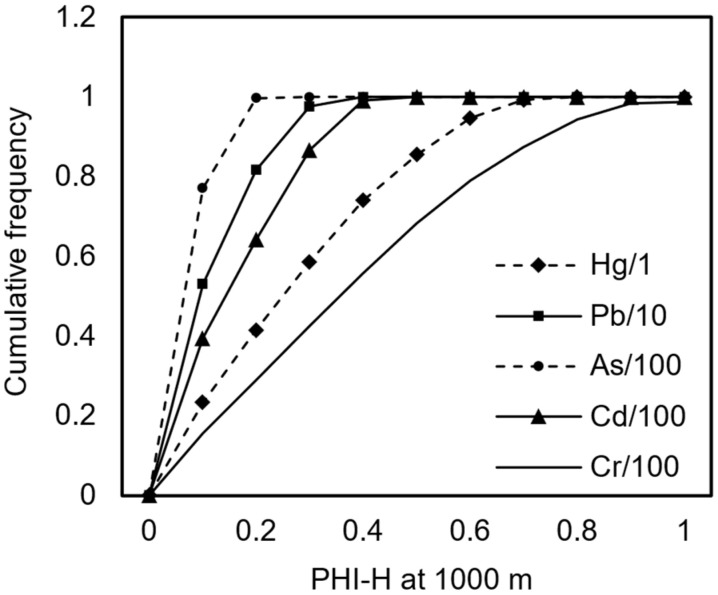
Cumulative frequency distribution curves of pollutant concentrations.

Based on the Monte Carlo simulation and uncertainty analysis methods, this study systematically assessed the diffusion characteristics of heavy metals from stockpile dust at a downwind distance of 1000 m (Figure 2). The simulation results showed that the concentration range of total suspended particulates was 1.2 to 11 µg/m^3^, significantly below the air quality standard of 80 µg/m^3^. However, the heavy metal pollution showed clear differentiation. Hg ranged from 0.005 to 0.047 µg/m^3^ and did not exceed the standard. Pb had an exceedance probability of 89.5%, with an exceedance multiple of 5.3. As ranged from 0.0077 to 0.14 µg/m^3^, Cd from 0.019 to 0.31 µg/m^3^, and Cr(VI) from 0.00024 to 0.0038 µg/m^3^. All had exceedance probabilities close to 100%, with exceedance multiples reaching 23.6 for As, 51.8 for Cd, and 131.5 for Cr(VI).

**Figure 3:**
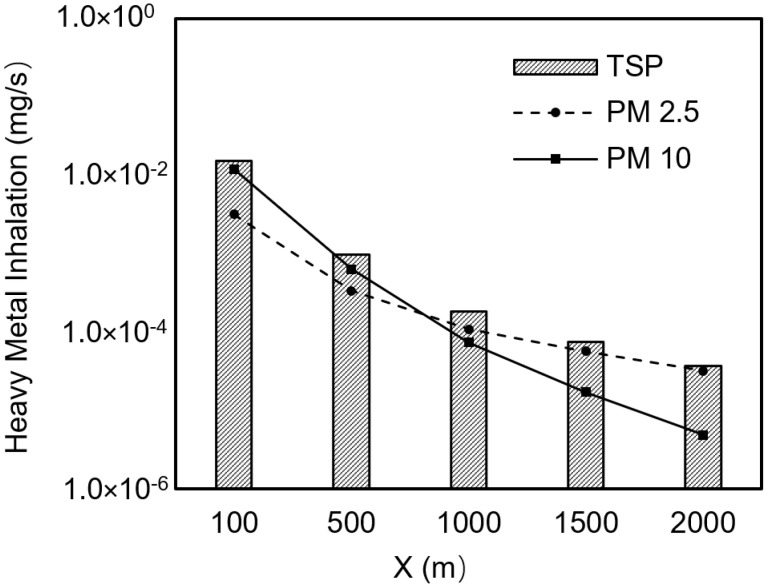
Variation of heavy metal exposure concentrations in dust with distance.

The migration and dispersion of dust in the atmosphere significantly affect the environmental quality of residential areas downwind. The study found that within a range of 100 to 2000 m from the stockpile, the concentration of particulate matter in the dust gradually decreased with increasing distance, but the concentration changes in the heavy metals carried by the dust were more complex (Figure 3). During the migration and dispersion of dust downwind, the concentrations of dust and heavy metals in the air changed rapidly. At a distance of approximately 180 m from the stockpile, the exposure concentration of PM10 dropped to 40 µg/m^3^, meeting the ambient air quality standard. However, the exceedance multiples of heavy metals in the air were extremely high at this point: Hg exceeded the standard by 16.2 times, Pb by 85 times, Cd by 631 times, As by 246 times, and Cr(VI) by 1677 times. When the distance increased to 450 m, the exposure concentration of PM2.5 dropped to 15 µg/m^3^, meeting the ambient air quality standard. At this point, the exceedance multiples of heavy metals in the air had decreased, with Hg exceeding the standard by 4.4 times, Pb by 18 times, Cd by 141 times, As by 96 times, and Cr(VI) by 488 times. Further research indicated that the compliance distances for heavy metals in dust to meet environmental quality control standards were 950 m for Hg, 2200 m for Pb, 4800 m for Cd, 4500 m for As, and 9400 m for Cr(VI). The particle size of dust also had a significant impact on its migration, dispersion, and pollution contribution. PM10 had a larger dust generation amount but settled quickly, affecting a relatively smaller area. In contrast, PM2.5 had a smaller dust generation amount but settled slowly, affecting a wider area. Moreover, PM2.5, with its smaller particle size and larger specific surface area, could adsorb more heavy metal pollutants, posing a greater environmental risk. For example, at a distance of 100 m from the stockpile, the contribution of PM10 to As exposure concentration was 79%, but as the distance increased, the contribution rate of PM10 gradually decreased. At 550 m, PM10 and PM2.5 each accounted for 50%, and at 2000 m, the contribution rate of PM2.5 reached 92%. These results indicate that although the concentration of particulate matter in the dust decreases with increasing distance, the environmental risk of heavy metal pollutants remains significant, especially at greater distances, where the role of PM2.5 is particularly prominent. This suggests that stricter monitoring and management measures are needed for areas surrounding stockpiles to ensure public health.

In the risk assessment of dust in residential areas located 500 m southwest of the stockpile, this study primarily considered the inhalation exposure pathway through the nasal cavity and calculated the carcinogenic risk and non-carcinogenic hazard quotient for cadmium, mercury, arsenic, and hexavalent chromium. According to the HJ 25.3 standard, a pollutant’s carcinogenic risk value exceeding 10^−6^ or a hazard quotient exceeding 1 is deemed unacceptable [16]. The calculation results showed that the concentrations of PM2.5 and PM10 in the residential area were 12 µg/m^3^ and 35 µg/m^3^, respectively, both meeting the ambient air quality standard limits. From Table 1, The specific risk assessment results indicated that the total carcinogenic risk value was 9.2 × 10^−7^, below the carcinogenic risk limit of 10^−6^, with PM10 contributing 66% of the risk. Arsenic had the highest carcinogenic risk value, at 4.8 × 10^−7^. The total non-carcinogenic hazard quotient was 0.15, below the hazard limit of 1, with cadmium contributing the most, with a non-carcinogenic hazard quotient of 0.11. This result is similar to the findings of Li et al. [21], who monitored and assessed the multi-dimensional spatial distribution of open-pit mine dust and found that heavy metals in dust pose a health risk to residents that cannot be ignored.

However, this study has certain limitations. First, it only considered the inhalation exposure pathway through the nasal cavity and did not include other possible exposure pathways such as ingestion and skin contact, which may underestimate the actual risk. Second, the meteorological conditions and dust activity intensity vary significantly across different seasons, leading to seasonal changes in dust risk [21]. This study did not conduct a detailed analysis of seasonal differences. Additionally, the risk of secondary dust resuspension after particle deposition was not considered, which may affect the assessment of long-term exposure risk.

### 3.3. Uncertainty and Sensitivity Analysis

Environmental risk assessment models face significant challenges due to the lack of uniformity and complexity of input parameters, which can lead to substantial discrepancies between model-predicted exposure concentrations and actual conditions [22]. Comprehensive field measurements, while more accurate, are costly and inefficient, and some parameters are difficult to obtain. Therefore, analyzing the sensitivity and uncertainty of model parameters is crucial to identify key parameters that significantly impact the simulation results, prioritizing them for field detection.

**Figure 4:**
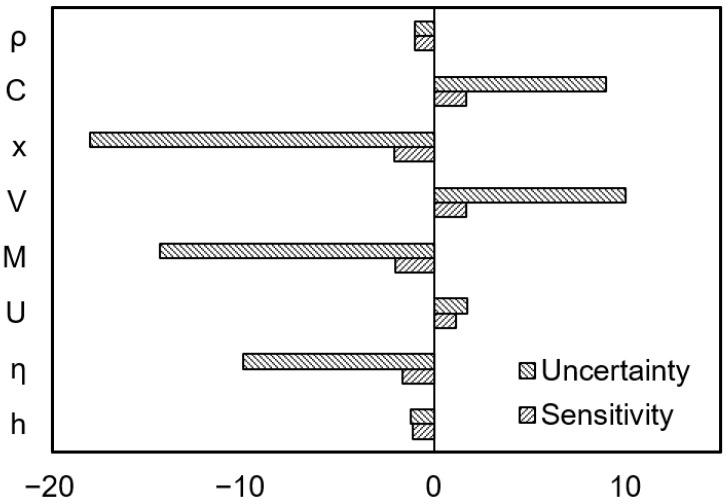
Sensitivity and uncertainty analysis.

Sensitivity analysis assesses the impact of parameters by adjusting them by ±25% of their default values, while uncertainty analysis quantifies the model prediction uncertainty by setting reasonable ranges for parameters. Both approaches ensure the effectiveness and reliability of the model. Figure 4 presents the sensitivity and uncertainty analysis results for the entire process of dust diffusion from the stockpile to residential areas via atmospheric dispersion. The analysis shows that during the pollutant emission stage, the moisture content of the stockpile has relatively high sensitivity, at 2.1 times, while the dust particle density has low sensitivity, at approximately 1.0 times, indicating minimal impact on heavy metal emissions from dust. When considering parameter uncertainty, the disturbance rate of the stockpile can cause a 10-fold difference in exposure concentration. During the pollutant migration and dispersion stage, the distance to the exposure point, dust removal rate, and wind speed have high sensitivity, at 2.0, 1.6, and 1.2 times, respectively. When considering parameter uncertainty, the impact of distance is particularly significant, with an uncertainty of 18 times, indicating that large solid waste stockpiles near residential areas should be phased out as soon as possible.

Sensitivity analysis reveals that the moisture content of the stockpile, dust removal rate, and distance are key factors in stockpile environmental risk assessment. Their uncertainties significantly affect the risk assessment results. Therefore, when conducting risk assessments and developing dust control strategies and interventions for stockpiles, it is essential to consider the impact of these parameters comprehensively. Special attention should be paid to the total amount of heavy metals in dust, their different chemical forms, and the toxicity of bioavailability, which pose potential threats to environmental safety. This enables more accurate assessments and refined management. The application of Monte Carlo simulation provides a more comprehensive and probabilistic approach to pollution risk assessment by considering the variability and uncertainty of input parameters. Compared with traditional deterministic pollution risk assessments, the probabilistic pollution risk assessment coupled with Monte Carlo simulation effectively reduces uncertainties caused by limited sample data. It provides quantitative indicators for the uncertainty and variability of stockpile dust risks, helping to gain a comprehensive understanding of the overall regional environmental pollution risks and offering more detailed and comprehensive information for scientific research and risk management.

## 4. Conclusions

This study conducted an in-depth analysis of the environmental risk characteristics of dust from magnesium slag stockpiles and its potential impact on surrounding residential areas. The findings indicate that during the open-air storage of magnesium slag, the pulverization process generates dust in which heavy metals (such as Pb, Cd, As, and Cr(VI)) are significantly enriched in PM2.5 compared to PM10. Within a downwind distance of 1000 m, the exceedance multiples for Cr(VI), As, and Cd are extremely high, reaching 131.5, 23.6, and 51.8, respectively, highlighting the significant impact on ambient air quality. Although the total carcinogenic risk (9.2 × 10^−7^) and the total non-carcinogenic hazard quotient (0.15) in residential areas did not exceed the limits, the risk contributions of As and Cd were relatively high, indicating that their potential hazards still require attention. Sensitivity analysis further revealed that the moisture content of the stockpile, dust removal rate, and distance are key factors influencing the environmental risk of dust. The uncertainties associated with these parameters have a significant impact on the risk assessment results. Therefore, it is recommended to increase the moisture content of the stockpile, optimize dust removal measures (such as installing windbreaks and dust suppression nets), and plan the location of stockpiles more reasonably to effectively reduce the pollution risks to the surrounding environment and public health posed by dust. Additionally, promoting the resource utilization of magnesium slag to reduce stockpile volumes is fundamental to solving dust-related issues.

## Figures and Tables

**Table 1:** Characterization of dust pollution risk.

	CR of PM2.5	HQ of PM2.5	CR of PM10	HQ of PM10
Cd	8.74 × 10^−8^	2.95 × 10^−2^	2.31 × 10^−7^	7.81 × 10^−2^
Hg	-	2.70 × 10^−4^	-	3.68 × 10^−4^
As	1.49 × 10^−7^	1.40 × 10^−2^	3.45 × 10^−7^	3.25 × 10^−2^
Cr(VI)	3.35 × 10^−8^	1.70 × 10^−4^	5.41 × 10^−8^	2.74 × 10^−4^
Total	2.70 × 10^−7^	4.40 × 10^−2^	6.31 × 10^−7^	1.11 × 10^−1^

## Data Availability

The data that support the findings of this study are available from the corresponding author upon reasonable request.
